# Supplementary material for: How the Severity and Mechanism of Recurrent Laryngeal Nerve Dysfunction during Monitored Thyroidectomy Impact on Postoperative Voice
Source: Cancers (Basel). 2021 Oct 27;13(21):5379. doi: 10.3390/cancers13215379 (PMC8582531; doi:10.3390/cancers13215379)
Supplement: Supplementary file 1 [file cancers-13-05379-s001.zip › cancers-1426561-supplementary.pdf]

cancers

MDPI

Supplementary Materials: How the Severity and Mechanism of Recurrent Laryngeal Nerve dysfunction during Monitored Thyroidectomy impact on Postoperative Voice

Tzu-Yen Huang, Wing-Hei Viola Yu, Feng-Yu Chiang, Che-Wei Wu, Shih-Chen Fu, An-Shun Tai, Yi-Chu Lin, Hsin-Yi Tseng, Ka-Wo Lee and Sheng-Hsuan Lin

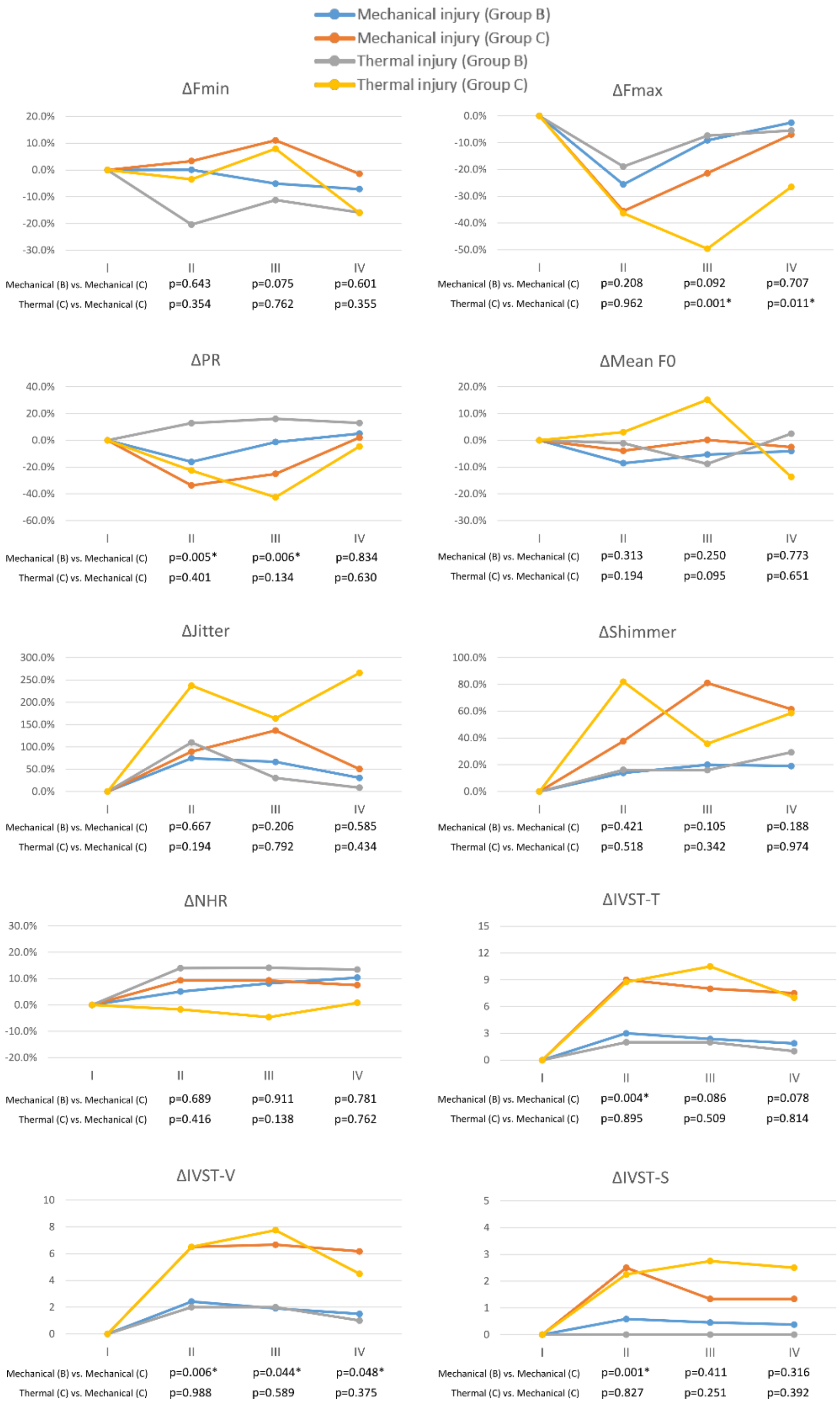

Figure S1. Voice parameter changes (Δ) with different mechanisms and severity of recurrent laryngeal nerve (RLN) dysfunction. Group B and C had post-dissection R2 signal decreases of 50–90% and >90%, respectively, from pre-dissection R1 signal. Blue line = Mechanical injury (Group B); Red line = Mechanical injury (Group C); Gray line= Thermal injury (Group B); Yellow line = Thermal injury (Group C). Abbreviations for objective/subjective voice parameters, the equation for calculating postoperative change in objective/subjective voice analysis, and the definition of follow-up periods (I/II/III/IV) are identical to those in Figure 3. The two statistical comparisons were (1) mechanical injury in Group B vs. mechanical injury in Group C; (2) thermal injury in Group C vs. mechanical injury in Group B. A p value <0.05 was considered statistically significant. Statistical analysis was not performed with thermal injury in Group B (Gray line) due to only one patient. * represents p value < 0.05, showed significant difference.

Table S1. Index of voice and swallowing handicap of thyroidectomy (IVST).

	**Questions**	**Never (0 point)**	**Sometimes (1 point)**	**Always (2 points)**
	Voice domain			
1.	My overall voice quality is abnormal.	0	1	2
2.	My voice difficulties restrict personal and social life.	0	1	2
3.	I feel my voice is hoarse.	0	1	2
4.	I feel as though I have to strain to produce voice.	0	1	2
5.	The sound of my voice varies throughout the day.	0	1	2
6.	I find it difficult to make a high-pitched voice.	0	1	2
7.	I find it difficult to make a low-pitched voice.	0	1	2
		IVST-V = ______ (Range from 0–14)		
	Swallowing domain			
8.	I feel strained when I speak or swallow.	0	1	2
9.	I choke when I drink (water or tea).	0	1	2
10.	I choke when I eat.	0	1	2
		IVST-S = ______ (Range from 0–6)		
	Total score	IVST-T = ______ (Range from 0–20)		

Table S2. Comparison of voice parameters of Group A, B and C in each follow-up period.

Parameters	Period	Group A	Group B	Group C	*p* value A vs. B	*p* value A vs. C	*p* value B vs. C
Fmin (Hz)	I	124.9 ± 31.9	128.4 ± 33.5	130.1 ± 28.7	0.436	0.387	0.189
	II	122.1 ± 38.5	125.5 ± 37.1	131.2 ± 35.8	0.520	0.602	0.494
	III	122.9 ± 51.3	116.3 ± 31.9	138.7 ± 45.2	0.406	0.500	0.301
	IV	127.0 ± 33.2	120.0 ± 21.8	122.5 ± 33.7	0.312	0.586	0.777
Fmax (Hz)	I	697.2 ± 385.7	599.6 ± 215.7	706.6 ± 243.8	0.206	0.898	0.667
	II	484.9 ± 180.6	429.5 ± 159.6	420.5 ± 170.9	0.077	0.160	0.807
	III	584.5 ± 357.8	494.1 ± 202.5	481.4 ± 208.8	0.297	0.574	0.818
	IV	605.9 ± 226.0	541.0 ± 223.8	546.2 ± 194.4	0.515	0.857	0.938
PR (Semitone)	I	29.4 ± 11.7	26.1 ± 7.2	28.5 ± 6.6	0.139	0.700	0.151
	II	23.6 ± 6.8	21.1 ± 6.8	19.5 ± 7.5	0.030 *	0.001 *	0.852
	III	26.3 ± 7.1	23.9 ± 6.6	20.9 ± 11.2	0.117	0.003 *	0.347
	IV	26.5 ± 6.9	24.9 ± 6.6	25.4 ± 6.3	0.825	0.525	0.811
Mean F0 (Hz)	I	192.8 ± 40.5	191.7 ± 39.5	202.5 ± 40.0	0.850	0.638	0.765
	II	182.2 ± 42.7	176.6 ± 47.9	197.1 ± 47.0	0.350	0.192	0.109
	III	187.6 ± 41.7	181.0 ± 42.5	203.4 ± 50.3	0.969	0.290	0.148
	IV	190.9 ± 37.4	190.3 ± 36.1	189.8 ± 54.5	0.939	0.911	0.974
Jitter (%)	I	1.61 ± 1.03	1.52 ± 1.03	1.61 ± 0.95	0.565	0.835	0.853
	II	1.87 ± 1.06	1.91 ± 1.33	2.77 ± 1.76	0.980	<0.001 *	<0.001 *
	III	1.61 ± 0.97	1.81 ± 1.29	2.72 ± 1.16	0.239	<0.001 *	0.057
	IV	1.76 ± 1.00	1.56 ± 1.48	2.35 ± 1.33	0.340	0.048 *	0.010*
Shimmer (%)	I	3.33 ± 2.09	3.51 ± 2.13	3.08 ± 1.50	0.545	0.481	0.313
	II	3.44 ± 3.31	3.79 ± 3.85	4.08 ± 3.84	0.513	0.946	0.706
	III	3.19 ± 2.19	3.79 ± 4.25	4.60 ± 3.47	0.130	0.040 *	0.607
	IV	3.49 ± 1.91	3.63 ± 2.67	4.16 ± 2.23	0.892	0.473	0.693
NHR (value)	I	0.129 ± 0.035	0.129 ± 0.036	0.129 ± 0.024	0.908	0.974	0.918
	II	0.131 ± 0.042	0.133 ± 0.043	0.132 ± 0.040	0.593	0.988	0.750
	III	0.126 ± 0.035	0.130 ± 0.035	0.128 ± 0.037	0.674	0.677	0.917
	IV	0.133 ± 0.042	0.132 ± 0.025	0.133 ± 0.031	0.934	0.863	0.783
IVST-T (score)	I	0.7 ± 1.7	0.7 ± 1.5	0.4 ± 1.2	0.981	0.452	0.583
	II	3.2 ± 2.9	3.8 ± 3.6	9.3 ± 3.1	0.582	<0.001 *	<0.001 *
	III	2.6 ± 3.0	2.7 ± 3.2	9.4 ± 5.7	0.882	<0.001 *	<0.001*
	IV	2.6 ± 2.8	2.9 ± 3.3	7.7 ± 6.0	0.824	0.025 *	0.046 *
IVST-V (score)	I	0.4 ± 1.1	0.4 ± 1.1	0.2 ± 0.6	0.995	0.307	0.542
	II	2.4 ± 2.3	2.9 ± 3.0	6.7 ± 2.4	0.565	<0.001 *	<0.001 *
	III	1.9 ± 2.3	2.3 ± 2.7	7.3 ± 4.0	0.466	<0.001 *	<0.001 *
	IV	1.9 ± 2.4	2.0 ± 2.5	5.7 ± 4.5	0.948	0.026 *	0.043 *
IVST-S (score)	I	0.3 ± 0.9	0.3 ± 0.7	0.2 ± 0.6	0.955	0.651	0.761
	II	0.9 ± 0.9	0.9 ± 0.9	2.6 ± 1.0	0.837	<0.001 *	<0.001 *
	III	0.7 ± 1.0	0.5 ± 0.9	2.1 ± 2.0	0.426	0.045 *	0.031 *
	IV	0.7 ± 0.8	0.9 ± 1.7	2.0 ± 2.0	0.734	0.061	0.119

Period I = Preoperative period (within 2 months before surgery); Period II = Immediate postoperative period (median duration of 3 days; range of 1–7 days); Period III = Short-term postoperative period (median duration of 12 days; range of 7–30 days); Period IV = Long-term postoperative period (median duration of 40 days, range of 30–90 days). * p value < 0.05, showed significant difference.
